# Comprehensive analysis of m6A modification in lipopolysaccharide-induced acute lung injury in mice

**DOI:** 10.1186/s10020-024-00782-2

**Published:** 2024-01-22

**Authors:** Chenzhen Xu, Congkuan Song, Wenjie Wang, Bohao Liu, Guorui Li, Tinglv Fu, Bo Hao, Ning Li, Qing Geng

**Affiliations:** 1 Department of Thoracic Surgery, Renmin Hospital of Wuhan University, Wuhan, 430060 China; 2 Department of Thoracic Surgery, The First Hospital of Jilin University, Changchun, 130021 China

**Keywords:** m6A methylation, MeRIP-Seq, ALI, METTL3, FTO

## Abstract

**Background:**

N6-Methyladenosine (m6A) methylation is the most prevalent post-transcriptional modification in mRNA, and plays significant roles in various diseases. Nevertheless, the precise functions of m6A modification in the formation of ALI remain unclear. In this study we explore the transcriptome distribution of m6A methylation and its probable roles of in ALI.

**Methods:**

Lipopolysaccharide (LPS) was utilized to establish an ALI mouse model. Real-time qPCR, Western blotting and m6A dot blot were utilized to assess m6A methylation level and the expression of m6A methylation enzymes. MeRIP-Seq and RNA-seq were utilized to explore differential m6A modifications and differentially expressed genes in ALI mice. The hub genes and enriched pathways were assessed by Real-time qPCR and Western blotting.

**Results:**

Our findings showed that overall m6A methylation level was increased in ALI mice lung tissues, accompanied by lower levels of METTL3 and FTO. Notably, the protein expression of these methylases were different in various cells. There were 772 differently expressed m6A peaks in ALI as compared to the control group, with 316 being hypermethylated and 456 being hypomethylated. GO and KEGG analyses demonstrated these differentially methylated genes were associated with the calcium signaling pathway and cAMP signaling pathway. Furthermore, we identified 50 genes with distinct m6A peaks and mRNA expressions by combined analysis of MeRIP-Seq and RNA-Seq. KEGG analysis also demonstrated that these overlapped genes were closely associated with the calcium signaling pathway, cGMP-PKG signaling pathway, etc. Besides, Western blotting results demonstrated that the protein expression of Fibronectin leucine-rich transmembrane protein 3 (Flrt3) as well as the calcium signaling pathway and cGMP-PKG signaling pathway, increased significantly after ALI.

**Conclusions:**

m6A modification was paramount in the pathogenesis of ALI, and provided a foundation for the further investigation in the prevention and treatment of ALI.

**Supplementary Information:**

The online version contains supplementary material available at 10.1186/s10020-024-00782-2.

## Background

N6-Methyladenosine (m6A) modification takes place at the N6 position of the adenine in RNA molecules, and is the most common post-transcriptional modification in eukaryotic mRNA (Elsabbagh et al. 2022; Luo et al. 2014). m6A was proven to take place at the consensus sequence RRACH (R = A/G, H = A/C/U)(Tan et al. 2022). It’s a reversible and dynamic process, regulated by m6A methyltransferase (writers), m6A demethylase (erasers) and m6A recognition factors (readers)(Rønningen et al. 2021). Writers catalyze the production of m6A on mRNA, which contain methyltransferase-like 3 (METTL3), METTL14, Wilms tumor 1-associating protein (WTAP) and other related proteins. Correspondingly, erasers can reduce m6A from methylated RNA, containing fat mass and obesity-associated protein (FTO) and alkB homolog 5 (ALKBH5). Readers recognize methylated m6A sites on RNA and affect RNA fate, which include YTH domain containing proteins 1/2 (YTHDC1/2), YTH-family proteins 1–3 (YTHDF1-3) and insulin-like growth factor 2 mRNA binding proteins 1–3 (IGF2BP1-3)(Huang et al. 2021; Tian et al. 2021; Wang et al. 2022b). Similar to other RNA post-transcriptional modification, m6A modification regulates key biological processes, including splicing, nuclear export, translation, mRNA stabilization, and mRNA degradation (Garbo et al. 2021; Valadon et al. 2021; Zhang et al. 2023). And m6A modification is considered to be closely correlated with a variety of physiological and pathological processes (Bu et al. 2023; Li et al. 2021; Wang et al., 2022c; Zhang et al., 2022d).

Acute lung injury (ALI) is a serious respiratory disorder featured by intractable hypoxemia and noncardiogenic pulmonary oedema, which is usually caused by bacterial or viral pneumonia, sepsis, major trauma and shock (Liu et al. 2021; Matthay et al. 2019). Acute respiratory distress syndrome (ARDS) is its more severe form. Uncontrolled inflammation response and alveolar/capillary barrier destruction are the central pathogenesis of ALI/ARDS (Matthay et al. 2019). Previous research has found that m6A methylation is crucial to various pathogenic processes of lung diseases. Zhang et al. demonstrated that in pulmonary fibrosis METTL3 can promote lung fibroblast-to-myofibroblast transition by regulating the methylation of KCNH6 mRNA (Zhang et al. 2021). Hu et al. showed that YTHDF1 can ameliorate pulmonary hypertension (PH) in mice through enhancing MAGED1 translation (Hu et al. 2021). In addition, a study from Chen et al. revealed that METTL3 inhibits endothelial barrier dysfunction and inflammatory response by targeting Trim59(Chen et al. 2022). However, the overall m6A methylation landscape of ALI has attracted little attention.

To explore the probable roles of m6A modification in ALI, meRIP-seq and RNA-seq were carried out to assess differential m6A peaks and mRNA expressions after ALI. We then identified the potential regulatory target by integrating MeRIP-seq and RNA-seq data. Overall, this study showed that m6A methylation modification could be an important intervention target in ALI.

## Materials and methods

### Animal and treatments

All experiment procedures followed the Guide for the Care and Use of Laboratory Animals by NIH, and were also authorized by the Animal Care and Use Committee of Renmin Hospital of Wuhan University (20,230,104 A). C57BL/6 male mice (8–10 weeks old) were furnished by the Hubei Province Experimental Animal Center (Wuhan, China). These mice were placed in the specific-pathogen-free (SPF) environment with temperature of 20–25℃ and humidity of 50 ± 5%. All mice were separated at random into 2 groups of 9 mice each. As described in our previous study (Ning et al. 2020), the LPS-induced ALI model was constructed by intratracheal instillation of LPS (Sigma-Aldrich, L2880) with a dosage of 5 mg/kg. Correspondingly, mice from the control group were instilled intratracheally with an isovolumetric sterile saline. Twelve hours later, these mice were sacrificed under deep anesthesia and both lungs were excised.

### Haematoxylin & eosin (H&E) staining

H&E staining was executed in the same manner as in our prior study (Ning et al. 2020). Left lungs were fixed in the 10% neutral buffered formalin before being embedded in paraffin, and sectioned transversely at 5 μm thickness. Sections were then stained with hematoxylin and eosin. A semi-quantitative scoring system was utilized to evaluate the severity of lung injury blindly by two independent pathologists, as described previously (Shi et al. 2021).

### Lung wet/dry ratio determination

After the surface blood had been wiped away, the weight of the right lung was measured. Following that, lung tissues were dried at 80 °C for 48 h to determine the dry weight. Subsequently, the wet/dry ratio was calculated.

### m6A dot blot

Total RNA was obtained from lung sample by the use of TRIzol (Invitrogen, 15,596,026) as directed by the manufacturer. Denatured RNA was spotted on the Hybond-N + membrane (Servicebio, G6018), and then cross-linked with UV. After incubation with anti-m6A antibody (Elabscience, E-AB-40,550) and rabbit-HRP secondary antibody (Proteintech, SA00001-2) in sequence, the membrane was reacted with ECL luminescent solution and was visualized by ChemiDoc system (Bio-Rad, USA).

### Real-time qPCR

cDNA Synthesis Kit (Servicebio, G3337-50) was utilized to synthesize cDNA. SweScript One-Step RT-PCR Kit (Servicebio, G3335-100) was then utilized for the quantitative real-time PCR, and the target RNA expression levels were standardized to GAPDH. The primers used were displayed in Table S1.

### Enzyme-linked immunosorbent assay (ELISA)

The concentrations of IL-1β, IL-6 and TNF-α in bronchoalveolar lavage fluid (BALF) were detected with commercial ELISA kits (Elabscience, China) following the manufacturer’s instructions. The absorbance was subsequent evaluated at 450 nm through a microplate reader.

### Cell culture and treatment

THP-1 cell line (The hominine monocytic cell), A549 cell line (lung epithelial cell) and HUVEC cell line (human umbilical vein endothelial cell) were all provided by the American Type Culture Collection (ATCC, Manassas, VA, USA). HPMEC cell line (human pulmonary microvascular endothelial cell) was obtained from Procell (Wuhan, China). The THP-1 cells were grown in RPMI 1640 culture medium (Servicebio, G4532-500ML), HUVECs in DMEM medium with high glucose (Servicebio, G4515-500ML), and A549 cells in Ham’s F-12 K medium (Servicebio, G4562-500ML). Each medium comprises 10% FBS (Gibco, USA) and 1% penicillin/streptomycin (Biosharp, China). HPMECs were cultured in microvascular endothelial cell growth medium (Procell, CM-H001). These cells were kept at a temperature of 37 degrees Celsius in the humidified incubator with 5% CO2. To obtain macrophage-like state, THP-1 cells were stimulated with 100 ng/ml phorbol 12-myristate 13-acetate (PMA; MCE) for 12 h. These THP-1 cells were cultured with LPS (1 µg/mL) for 12 h, while HUVEC, HPMEC and A549 cells were all cultured with LPS (10 µg/mL) for 12 h.

### Western blot

RIPA Lysis Buffer (Servicebio, G2002-100ML) with 1% Phenylmethanesulfonyl fluoride (PMSF; Servicebio, G2008-1ML) were used to lyse lung tissues and cells, and BCA assay kit (Servicebio, G2026-1000T) was utilized to determine the protein concentration. Proteins were separated on 10% SDS-PAGE gels at appropriate concentrations and then transferred onto nitrocellulose filter membranes. Following blocking with 5% skim milk for 2 h, these membranes were incubated overnight at 4 °C with primary antibodies against METTL3 (Baijia, BPB2248), FTO (Wanleibio, WL05010), CaMK II (Baijia, IPB1535), FLRT3 (Baijia, IPB7433), PKG (Baijia, IPB7812), p-CaMKII (ELK Biotechnology, ES7642) and GAPDH (Abcam, ab8245). Subsequently, following three washes with TBST, matching secondary antibodies (Abclonal, AS063, AS064) were used to incubate the blots for 2 h at the ambient temperature. At last, the enhanced chemiluminescence western blotting detection system were utilized to visualize protein bands.

### Immunohistochemical staining

Deparaffinized lung tissue sections were rehydrated and then exposed to heat induction antigen retrieval, followed by 3% H_2_O_2_ treatment to inactivate endogenous peroxidase. After blocking nonspecific binding sites of proteins with 10% goat serum for 1 h, the lung tissue slices were incubated at 4 °C with primary antibodies against METTL3 (1:50) and FTO (1:50). Next, biotinylated secondary antibody was used to incubated at 37 °C for 1 h. At last, these sections were treated for 5 min at the ambient temperature with diaminobenzidine before being examined under a light microscope.

### Cell viability

A CCK8 assay kit (GLPBIO, GK10001) was employed to assess cell viability according to the manufacturer’s protocol. On summary, after being placed on 96-well plates for 12 h, cells were incubated with different doses of LPS for 12 h before cell viability was determined.

### MeRIP-Seq and RNA-Seq

RNA samples from 3 ALI mice and an equivalent number of control group mice were used for MeRIP-Seq and RNA-Seq. The MeRIP-Seq and RNA-Seq service were provided by Seqhealth technology Co., Ltd. In brief, RNA was randomly fragmented into 100-bp segments, and subsequently immunoprecipitated with anti-m6A antibody (Synaptic Systems, 202,003) to acquire m6A-methylated RNA fragments. The first strand cDNA was synthesized after elution, and was amplified by PCR after adapter ligation to construct library. The Illumina NovaSeq instrument was used for the library sequencing.

### Sequencing data processing and bioinformatics analyses

Raw data were filtered with Cutadapt software (v2.4.0) to obtain high-quality clean data, which were subsequently aligned with the mice reference genome (GRCm38) with HISAT2 software (v2.1.0). We used MACS software to determine m6A peaks (m6A methylated sites on RNA) and analyzed the motifs of m6A peaks perform with HOMER software. Differential m6A peaks and mRNA were filtered with threshold of *p* value < 0.05,|fold change| > 2. And then they were carried out for Gene Ontology (GO) and Kyoto Encyclopedia of Genes and Genomes (KEGG) analyses by bioinformatics platform.

### Protein-protein interaction (PPI) network analysis

The genes with differential expression were uploaded to the STRING database in order to establish PPI network, with interaction score setting as medium confidence (0.400). Subsequently, these data were imported to Cytoscape 3.9.1 software to visualize the network, and then ranked by the degree method.

### Statistical analysis

Statistical analyses for this study utilized SPSS 21.0 and GraphPad Prism 8 software. Quantitative data are reported as mean ± standard deviation (SD). Differences between two groups were compared using an unpaired Student’s t-test. *P*-value<0.05 was considered statistically significant.

## Results

### Successful establishment of LPS- induced ALI mice model

**Fig. 1:**
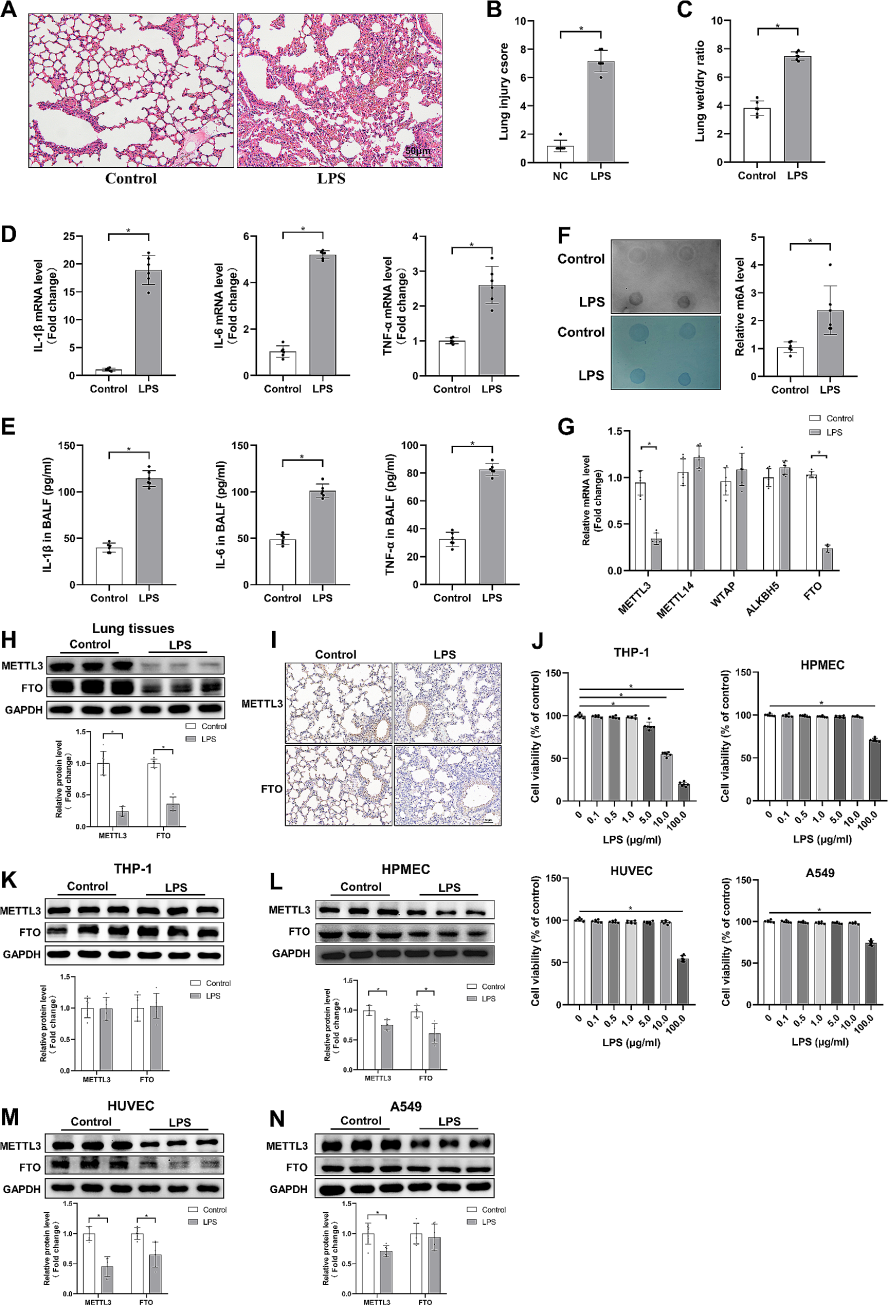
m6A modification level and methylase expression in ALI. (**A**, **B**) H&E staining of lung tissue after LPS treatment for 12 h and corresponding lung injury score (*n* = 6). (**C**) Lung wet/dry ratio (*n* = 6). (**D**) Relative mRNA levels of IL-1β, IL-6 and TNF-α in lung tissues (*n* = 6). (**E**) The concentrations of IL-1β, IL-6 and TNF-α in BALF tested by ELISA (*n* = 6). (**F**) Dot blot images of m6A methylation level in lung tissues and corresponding quantitative results (*n* = 6). (**G**) The relative mRNA levels of m6A methylation related genes in lung tissues (*n* = 6). (**H**) Western blots for METTL3 and FTO in lung tissues and quantitative outcomes (*n* = 6). (**I**) Immumohistochemical staining of METTL3 and FTO in lung tissues. (**J**) Cell viability measured by CCK8 kit in differentiated THP-1 cells, HPMECs, HUVECs and A549 incubated with different doses of LPS for 12 h (*n* = 6). (**K**-**N**) Western blots for METTL3 and FTO respectively in differentiated THP-1 cells, HPMECs, HUVECs and A549 cells, and corresponding quantitative outcomes (*n* = 6). (Values are presented as Mean ± SD, *p < 0.05)

To confirm successful establishment of ALI model, 12 h after intratracheal LPS instillation, lung pathological changes were observed by H&E staining. There was inordinate lung tissue structure, obvious inflammatory cell infiltration and severe interstitial edema in the LPS group as compared to the control group (Fig. 1A). What’s more, the lung injury score and wet/dry ratio were considerably increased in LPS group (Fig. 1B, C). LPS also caused lung inflammatory response, which was indicated by the increase mRNA levels of IL-1β, IL-6 and TNF-α (Fig. 1D). We also found that the concentrations of these inflammatory cytokines in BALF consistently increased following LPS treatment (Fig. 1E). These data suggested that we had successfully established the LPS-induced ALI mice model.

### Overall m6A methylation level in mice lung tissues

To detect whether m6A modification was altered in response to LPS stimulation, m6A dot blot analysis was performed next. As shown in Fig. 1F, Our data revealed that compared to the control group, m6A modification level was substantially increased after LPS stimulation (P<0.05). We next evaluated the mRNA levels of m6A modification-related proteins in lung tissues to illuminate the key modification enzymes by RT-qPCR analysis, including m6A methyltransferase (METTL3, METTL14 and WTAP) and m6A demethylase (ALKBH5 and FTO). Notably, METTL3 and FTO expression levels were considerably lower than the control group, but others revealed no statistically significant differences (Fig. 1G). We furthermore verified the protein expressions of METTL3 and FTO by Western blot, and in line with RT-qPCR results, the protein expressions of these genes were considerably reduced in ALI (Fig. 1H). Immunohistochemical staining also revealed consistent conclusions (Fig. 1I). To investigate the changes of m6A methylation enzymes in different cells, we further evaluated the protein expressions of METTL3 and FTO after LPS stimulation in four cell lines, which are of pivotal importance to the development of ALI. To rule out the possibility of potential bias produced by LPS toxicity on cell viability, we further investigated the effects of different LPS concentrations on the cell viability in different cell lines. Results demonstrated that a high dosage of LPS (100 µg/ml) inhibited the cell activity of HPMECs, HUVECs and A549 cells, whereas a lower dose of LPS (5 µg /ml) inhibited the cell activity of differentiated THP-1 cells (Fig. 1J). Therefore, combining previous researches, we decided to treat differentiated THP-1 cells with LPS at a concentration of 1 µg/ml, while HUVECs, HPMECs and A549 cells with LPS at a concentration of 10 µg/ml (Kang et al. 2022; Li et al. 2023b; Pasaoglu et al. 2014). As shown in Fig. 1K, the expressions of METTL3 and FTO didn’t change significantly in differentiated THP-1 cells. As for HPMECs and HUVECs, LPS stimulation significantly decreased the protein levels of METTL3 and FTO (Fig. 1L, M). Additionally, the protein level of METTL3 was declined but FTO expression level maintained unchanged in LPS-treated A549 cells (Fig. 1N). Overall, these data indicated that LPS stimulation increased the global m6A methylation levels in lung tissue, which might be attributed to decreased expression of METTL3 and FTO. Furthermore, the expression profiles of m6A methyltransferase and demethylase can be different among specific lung cells after ALI.

### Overview of the m6A methylation in ALI

**Fig. 2:**
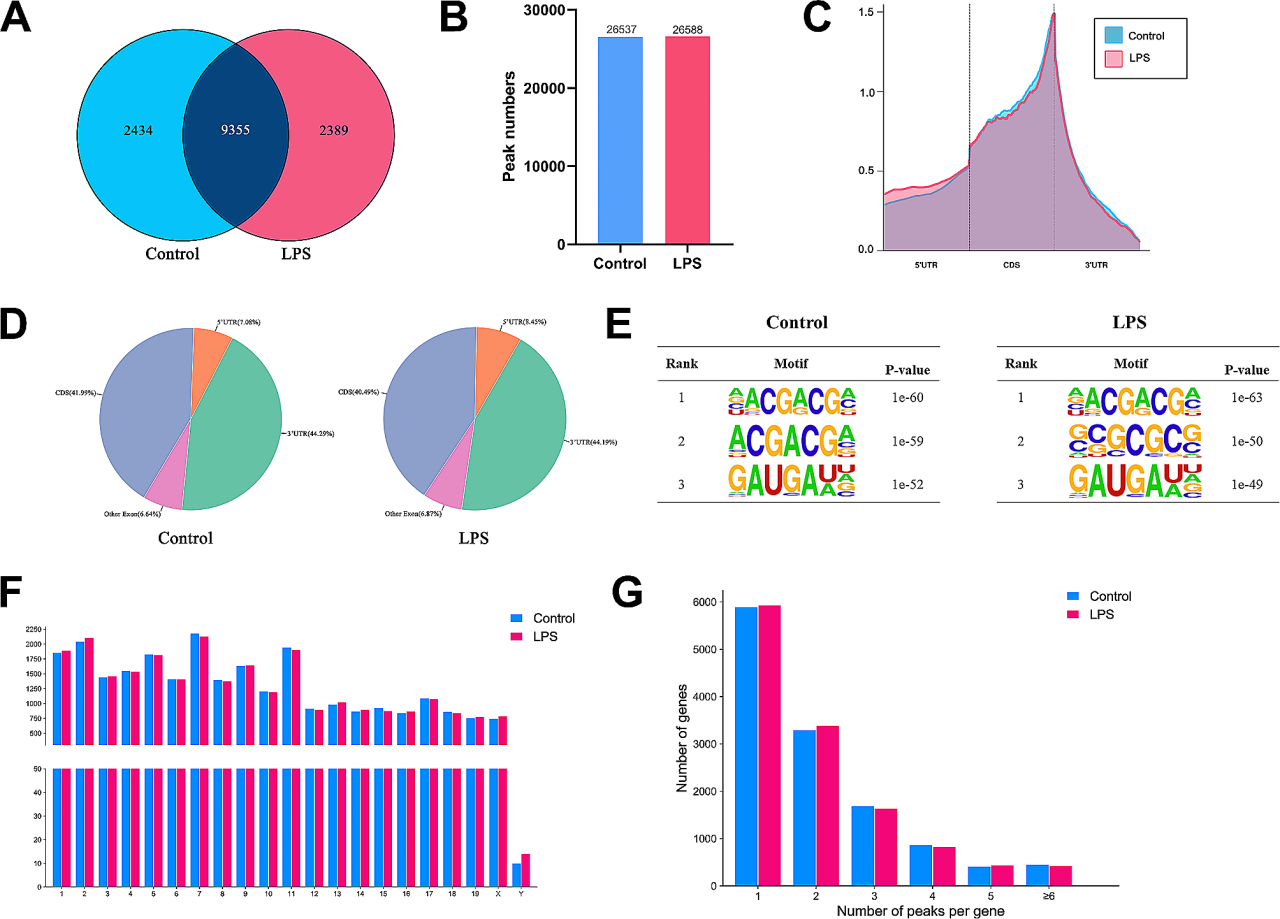
Overview of m6A modification map of two groups. (**A**) Venn diagram showed overlap of m6A modifier genes between two groups. (**B**) Total number of m6A peaks per group. (**C**, **D**) The m6A peak distributions in the transcripts of each group shown by density curve and Pie charts. (**E**) Top 3 m6A motifs enriched in two groups. (**F**) The quantity of m6A peaks mapped to each chromosome. (**G**) The count of m6A peaks in per gene

The transcriptome sequencing of m6A modification in lung tissues from the LPS and control groups was compared. There were 11,744 and 11,789 m6A modifier genes in LPS and control groups, respectively, with 9,355 genes consistent between the two groups (Fig. 2A). What’s more, MeRIP-seq analysis displayed that an average of 26,537 m6A peaks were identified in controls and 26,588 in the LPS group (Fig. 2B). Figure 2C, D showed the m6A peak distributions within the whole transcriptome. m6A methylation was primarily enriched in the 3′ untranslated regions (3′UTRs) and coding sequences (CDSs), where proportions of both groups were analogous. Our study subsequently identified the top three m6A motifs which were showed as the sequence logo, and the “NACGACGM” sequence was the most significant one (Fig. 2E). In addition, m6A peaks could be observed in all chromosomes, with the highest frequency on chr7, chr2, chr11, chr1 and chr5 (Fig. 2F). However, the number of m6A modification sites on chromosomes did not differ between both groups. In terms of the distribution of methylation sites in each gene, we noticed that about half of genes exhibited a single m6A peak, while approximately 90% of genes had 1–3 m6A peaks (Fig. 2G).

### Differential methylated m6A genes and biological functional

**Fig. 3:**
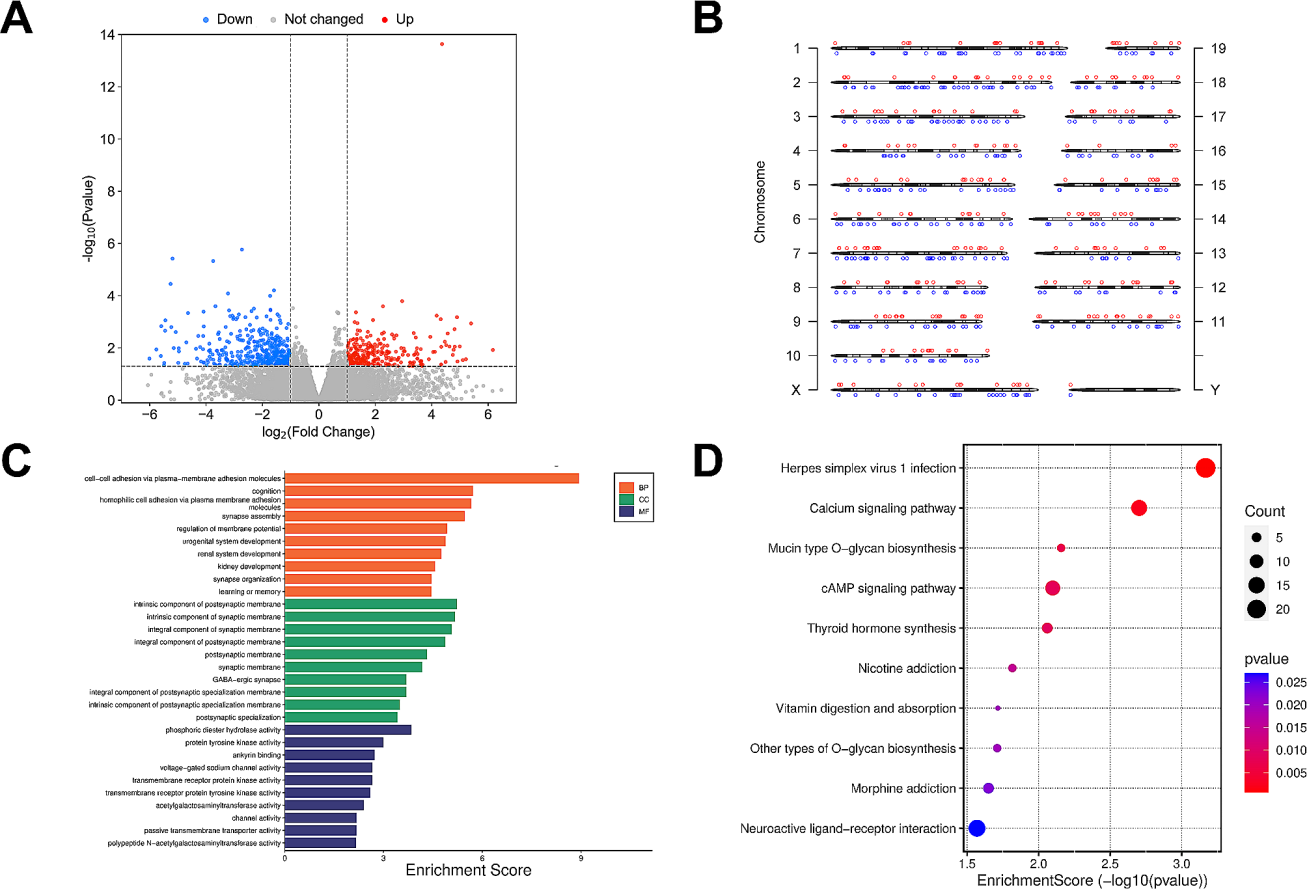
Differential m6A methylated genes in ALI. (**A**) Volcano plot showed the significantly changed m6A peaks between two groups. (**B**) The distribution of significantly differential m6A peaks across chromosomes. The red and blue dots to the top and to the bottom of each chromosome represent hypermethylated peaks and hypomethylated peaks respectively. (**C**) Top ten GO terms for genes with significantly changed m6A methylation. (**D**) Top ten enriched pathways for genes with changed m6A methylation

To further examine the alteration of m6A modification in ALI, we filtrated differential methylated genes using the *p* value < 0.05 and|fold change| > 2 criterion. Our data showed that there were 772 methylation sites in 657 genes were differently expressed after LPS stimuli, containing 316 hypermethylated sites and 456 hypomethylated sites (Fig. 3A). Moreover, Table 1 displays top ten hypermethylated and hypomethylated genes, and Table S2 lists all of the differentially methylated m6A genes. As shown in Fig. 3B, we labelled these altered gene distributions on the chromosomes respectively.

**Table 1:** Top 10 hypermethylated genes and hypomethylated genes

Name	Chr	Transcript ID	Peak Start	Peak End	Log2FC	*P*-value	Hyper/Hypo
Prss35	9	ENSMUST00000179574	86,743,648	86,743,709	6.168	0.012	Hyper
Tro	X	ENSMUST00000112709	150,657,296	150,657,387	5.395	0.001	Hyper
Sox6	7	ENSMUST00000206034	115,474,253	115,474,584	5.213	0.027	Hyper
Gm38313	3	ENSMUST00000191953	85,195,181	85,196,447	5.104	0.029	Hyper
Hrh3	2	ENSMUST00000056480	180,099,583	180,100,060	4.995	0.009	Hyper
Prune2	19	ENSMUST00000087689	17,123,279	17,123,430	4.914	0.033	Hyper
Dnajc6	4	ENSMUST00000106930	101,618,407	101,618,528	4.896	0.001	Hyper
Ighv5-2	12	ENSMUST00000195468	113,578,503	113,578,990	4.818	0.009	Hyper
Elovl4	9	ENSMUST00000034796	83,778,691	83,778,992	4.809	0.002	Hyper
Lrrc9	12	ENSMUST00000162159	72,476,158	72,477,319	4.778	0.012	Hyper
Prox1	1	ENSMUST00000175916	190,121,060	190,121,421	-6.006	0.026	Hypo
Ribc2	15	ENSMUST00000023067	85,144,480	85,144,569	-5.763	0.011	Hypo
Fam122b	X	ENSMUST00000071023	53,244,430	53,244,521	-5.603	0.020	Hypo
Usp51	X	ENSMUST00000095755	153,008,254	153,009,178	-5.587	0.001	Hypo
Ttn	2	ENSMUST00000011934	76,751,325	76,751,386	-5.492	0.046	Hypo
Ppp1r1c	2	ENSMUST00000090760	79,808,593	79,808,684	-5.483	0.038	Hypo
Saxo2	7	ENSMUST00000056728	82,632,959	82,633,109	-5.443	0.001	Hypo
Fat3	9	ENSMUST00000217308	16,375,131	16,375,701	-5.256	0.000	Hypo
Ccdc148	2	ENSMUST00000077687	58,822,144	58,822,295	-5.232	0.002	Hypo
Cldn2	X	ENSMUST00000135224	139,806,154	139,806,215	-5.188	0.000	Hypo

Simultaneously, GO and KEGG pathway analyses were used to clarify the biological function of differential methylated genes. Figure 3C showed the top ten enriched GO terms of genes with altered m6A peaks, containing cell-cell adhesion via plasma-membrane adhesion molecules, homophilic cell adhesion via plasma membrane adhesion molecules, phosphoric diester hydrolase activity, protein tyrosine kinase activity, etc. Similarly, KEGG analyses illuminated that differential methylated m6A genes were mostly abundant in the pathway including calcium signaling pathway, O-glycan biosynthesis and cAMP signaling pathway (Fig. 3D).

### Differential mRNA expression in ALI

**Fig. 4:**
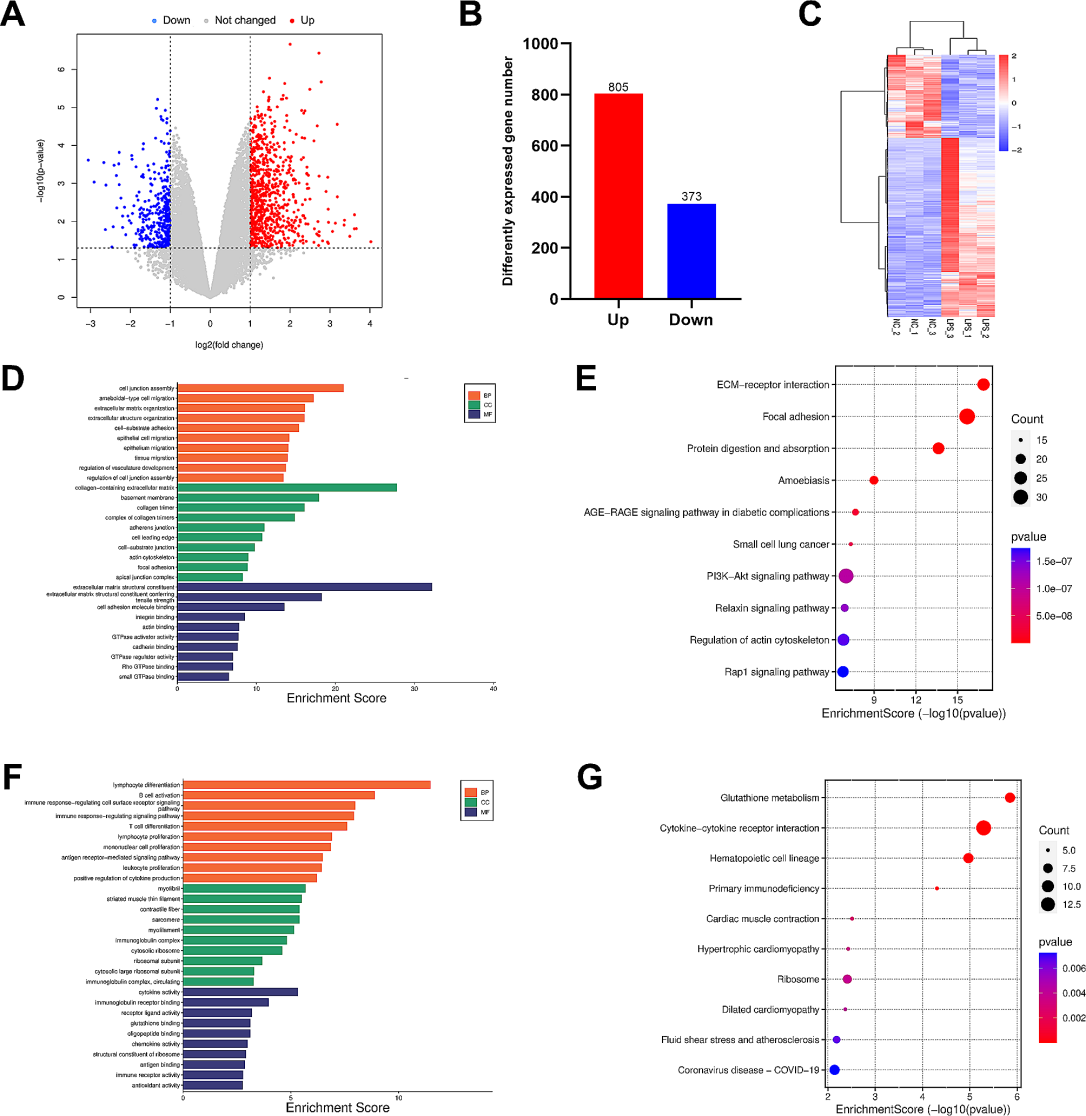
The overall expression of mRNA in ALI. (**A**) Volcano plot revealed the DEGs in ALI. (**B**) Number of upregulated and downregulated genes after ALI. (**C**) Heatmap showed the DEGs between the two groups. (**D**) Top ten GO terms of upregulated DEGs. (**E**) Top ten enriched pathways of upregulated DEGs. (**F**) Top ten GO terms of downregulated DEGs. (**G**) Top ten enriched pathways of downregulated DEGs

With the performance of RNA-Seq analysis, we generated a volcano plot of differentially expressed genes (DEGs), with 805 highly expressed genes and 373 low expressed genes (Fig. 4A, B). The heatmap showed the relative expression levels of each sample in control group and LPS group, accompanied by obvious different patterns between two groups (Fig. 4C). We also listed all upregulated and downregulated DEGs in the Table S3. GO and KEGG analyses were performed to better elucidate the function of these DEGs. GO analysis revealed that upregulated DEGs were closely related to cell junction assembly, extracellular matrix organization, epithelial cell migration in biological process, and collagen-containing extracellular matrix, basement membrane, adherens junction in cellular component, while extracellular matrix structural constituent, cell adhesion molecule binding, actin binding in molecular function (Fig. 4D). The downregulated DEGs were highly enriched in B cell activation, immune response-regulating signaling pathway, leukocyte proliferation in biological process, and myofibril, immunoglobulin complex, cytosolic ribosome in cellular component, while cytokine activity, chemokine activity, immune receptor activity in molecular function (Fig. 4F). As for KEGG analysis, our data revealed that upregulated DEGs were highly linked to ECM-receptor interaction, focal adhesion, PI3K-Akt signaling pathway, regulation of actin cytoskeleton and Rap1 signaling pathway (Fig. 4E), whereas downregulated DEGs in glutathione metabolism, cytokine-cytokine receptor interaction and coronavirus disease - COVID-19 (Fig. 4G).

### Conjoint analysis of MeRIP-Seq and RNA-Seq after ALI

**Fig. 5:**
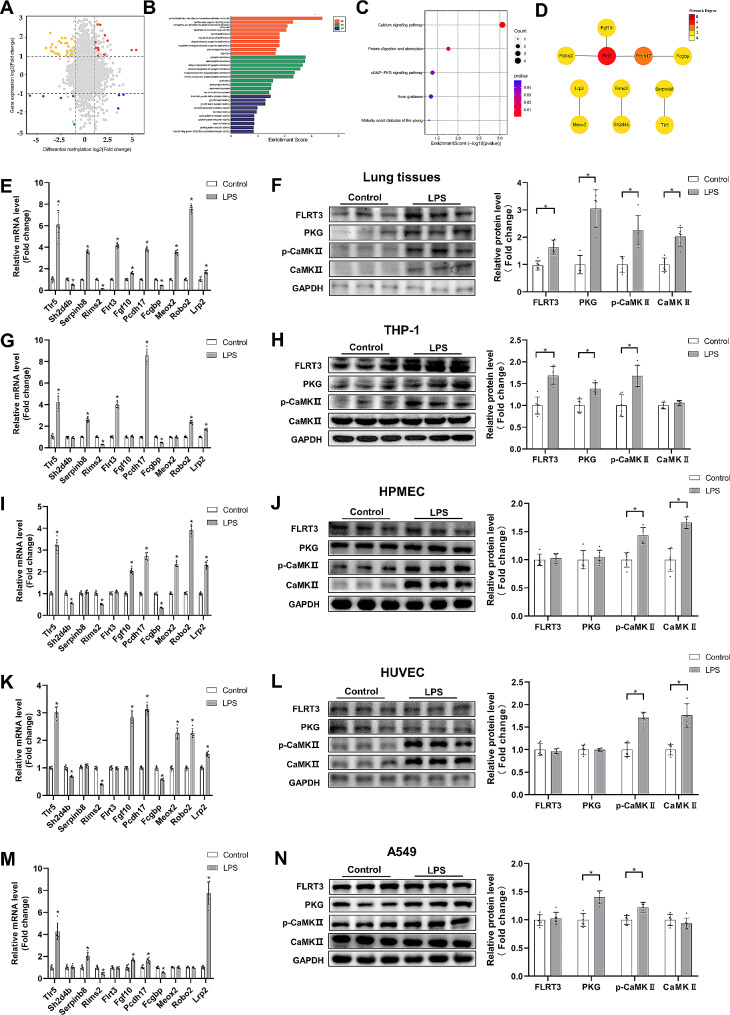
Conjoint analysis of m6A methylation and mRNA expression. (**A**) Four-quadrant plot showed the genes with both differential m6A peaks and concordant mRNA expression changes. (**B**) GO terms for the overlapped genes. (**C**) Enriched pathways for the overlapped genes. (**D**) PPI network analysis for the overlapped genes. (**E**, **F**) Relative mRNA levels of eleven hub genes and western blots for Flrt3, PKG, CaMKII and p-CaMKII in lung tissues and quantitative outcomes (*n* = 6). (**G**-**N**) Relative mRNA levels of eleven hub genes and western blots for Flrt3, PKG, CaMKII and p-CaMKII respectively in differentiated THP-1 cells, HPMECs, HUVECs and A549 cells, and corresponding quantitative outcomes (*n* = 6). (Values are presented as Mean ± SD, *p < 0.05)

Using a combined analysis of MeRIP-Seq and RNA-Seq, the genes with differential m6A peaks and mRNA expressions were split into four groups and visualized in a four-quadrant graph (Fig. 5A). With the filtering settings of *p* value < 0.05 and|fold change| > 2, 50 genes were identified to be eligible. Among these genes, there were 13 hypermethylated genes upregulated, 3 hypermethylated genes downregulated, 29 hypomethylated genes upregulated, 5 hypomethylated genes downregulated. GO analysis demonstrated that these genes were associated with cell-cell adhesion via plasma-membrane adhesion molecules, fat cell proliferation, fibroblast growth factor receptor binding, transforming growth factor beta-activated receptor activity, etc. (Fig. 5B). Moreover, KEGG analysis demonstrated that there were 5 pathways markedly enriched, including calcium signaling pathway, protein digestion and absorption, cGMP-PKG signaling pathway (Fig. 5C). The PPI network analysis was also performed to construct the interaction networks. Fibronectin leucine-rich transmembrane protein 3 (Flrt3) and Protocadherin 17 (Pcdh17), As seen in Fig. 5D, were essential components of the entire PPI network.

To further verify the expression of hub genes and pathways filtered after comprehensive analysis, we identified the mRNA levels of hub genes in lung tissues through RT-qPCR analysis. As shown in Fig. 5E, the mRNA levels of hub genes had altered significantly, and their trends were consistent with the RNA-seq results. In addition, the protein expression of Flrt3 was significantly enhanced in the lung tissues of ALI mice, which was in line with RT-qPCR data (Fig. 5F). Given that Ca^2+^/calmodulin-dependent protein kinase II (CaMKII) is activated by an increase in intracellular Ca^2+^, we selected CaMKII and its phosphorylated form as the target for calcium signaling pathway activation (Irnaten et al. 2020; Jiang et al. 2020). Our data showed that calcium signaling pathway and cGMP-PKG signaling pathway were both enhanced in LPS-induced ALI mice model (Fig. 5F). To further investigate whether these possible hub genes and enriched pathways were differentially expressed in specific lung cells, we validated them in different cell lines. As for eleven hub genes, they were observed to be, at least in part, differentially expressed in differentiated THP-1 cells, HMPECs, HUVECs and A549 cells, respectively (Fig. 5G, I, K, M). The protein level of Flrt3 was only elevated in LPS-induced THP1 cells, but had no obvious changes in epithelial cells or endothelial cells (Fig. 5H, J, L, N). Moreover, the calcium signaling pathway was activated in each cell lines after LPS incubation, whereas the cGMP-PKG signaling pathway was stimulated only in THP-1 cells and A549 cells, but not in HMPECs nor HUVECs (Fig. 5H, J, L, N).

## Discussion

**Fig. 6:**
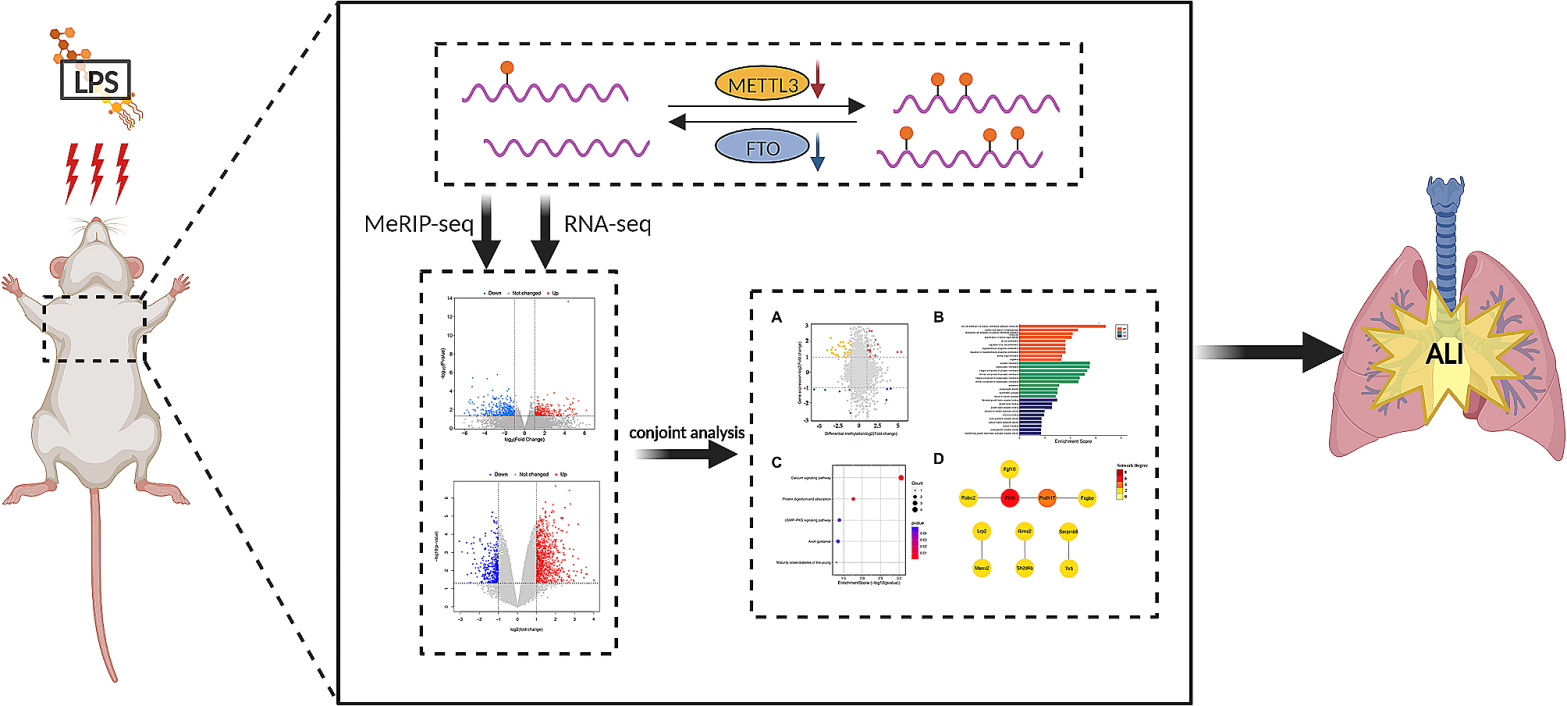
Graphical abstract of the possible mechanism by which m6A methylation participates in ALI

m6A modification, as the most common RNA modification in eukaryotes, is closely linked to the pathogenesis of diverse diseases, particularly in many lung diseases. However, there are currently scarcely any studies on the transcriptome-wide distribution and the expressed differences of m6A in LPS-induced ALI. Herein, we innovatively investigated the overall changes and crucial function of m6A methylation in ALI, and further proposed a novel understanding of pathogenesis in LPS-induced ALI (Fig. 6).

In this study, we reported that m6A methylation modification shifted considerably during ALI. The dot blot data demonstrated that the overall m6A modification level rose in ALI, reflecting the significance of m6A modification in affecting the process of ALI. Given that m6A methylation of RNA is a reversible and dynamic process, we investigated the expression levels of related m6A methylases in lung tissues. Interestingly, not only the expression of demethylase FTO was reduced in ALI, but so was the expression of methyltransferase METTL3. Recent studies have revealed that m6A modification was able to enhance ALI. Neutrophil extracellular traps (NETs), which was highly expressed in ALI patients and sepsis-induced ALI mice models, were found to increase m6A modification level, hence resulting in ferroptosis and impaired autophagic flux by targeting HIF-1α and Sirt1 in alveolar epithelial cells, suggesting significant roles RNA m6a methylation playing in ALI (Qu et al. 2022; Zhang et al. 2022a). Notably, these studies identified overexpressed METTL3 as the major regulator to the elevation of m6A methylation. Although we found global m6A level was raised consistently, METTL3 was observed to be lower inversely in our research, which might due to the difference in ALI modeling approaches. Since above researches on m6A methylation were at the cellular level rather than at the overall level of lung tissue, we further explored whether this difference was driven by differential expression of methylases in distinct cells. Our results shown that METTL3 decreased whereas FTO remained unchanged in alveolar epithelial cells. Furthermore, METTL3 and FTO were both reduced in HUVECs while remaining intact in macrophages. Chen et al. revealed that decreased expression of METTL3 accelerated vascular endothelial barrier dysfunction and inflammatory responses by affecting the stability Trim59 mRNA, which was consistent to our findings (Chen et al. 2022). Taken together, these results suggest that the progression of ALI may be driven by an intricate comprehensive outcome of methyltransferase METTL3 and demethylase FTO mediating the m6A modification of different RNAs in specific lung cells. Given the upregulation in the global m6A methylation level in ALI, we dare to hypothesize that m6A modification levels in lung tissues involve the integrated regulation of various methylases in different cells. And in ALI, the global m6A levels and the expressions of methylases in different cells may be varied or even opposite.

Herein, we first performed MeRIP-seq and RNA-seq in lung tissues from mice with ALI and control mice to characterize m6A methylation across the transcriptome. We found a total of 772 significantly changed peaks between the two groups, of which 316 hypermethylated and 456 hypomethylated peaks were detected, implying that changes of m6A modification were related to the process of ALI. In addition, the distribution of m6A peaks was primarily found in the 3′UTR and CDS regions, which was in accordance with other sequencing studies of lung diseases (Hu et al. 2023; Zhang et al., 2022c). Methylation levels at these regions are related to the biological functions of mRNA including stability, translation and selective polyadenylation (Wang et al. 2014; Yue et al. 2018). Meanwhile, almost half of methylation transcripts contained one m6A peak, while approximately 25% containing three or more m6A peaks. Notably, in despite of the differences in m6A peaks and methylated genes, the distribution of m6A modification within transcripts was analogous between the groups. In summary, these data imply m6A methylation may be crucial to ALI.

We further carried out GO and KEGG analyses to better understand the biological functions of differentially methylated genes in ALI. We discovered that these genes were primarily abundant in the calcium signaling pathway, mucin type O-glycan biosynthesis and cAMP signaling pathway. Intracellular calcium is a key second messenger, and the calcium signaling pathway serves an assortment of physiological processes such as cell differentiation, proliferation, motility, apoptosis, and so on (Dewenter et al. 2017). As a ubiquitous intracellular second messenger molecule, cAMP is of crucial importance to various of physiological and pathological processes (Aslam et al. 2022). cAMP has long been considered to be an inducer of anti-inflammatory responses (Tavares et al. 2020). In fact, multiple investigations have demonstrated that the cAMP signaling pathway in many cells had a role in controlling LPS-induced ALI (Duan et al. 2022; Wang et al. 2022a). However, it still remains to be explored whether these pathways are modified by m6A methylation.

m6A modification participates in regulating the fates of modified RNAs and affecting gene expression (Huang et al. 2021). By integrating MeRIP-Seq and RNA-seq data, we identified 50 genes in ALI that exhibited both differential m6A peaks and synchronized changes in mRNA expression. The expression of these genes might be controlled by post-transcriptional modification of m6A methylation. Among these, a few genes have been reported to be associated with ALI, such as toll-like receptor 5 (TLR5)(Hu et al. 2012). PPI network analysis further revealed that Flrt3 was located at the core position of the whole regulatory pathway, and the protein expression of Flrt3 was observed increased in ALI mice lung tissue and LPS-stimulated macrophages (Fig. 5F, H). Flrt3 has been reported to suppress epithelial-mesenchymal transition (EMT) and promote apoptosis in colorectal cancer cells (Yang et al. 2022). However, the precise function of Flrt3 in ALI still remains to be further explored, and its potential function in macrophages deserves attention. Given that some of the genes were not proved to be associated with ALI, we further focused on the relevant signaling pathways. Our data demonstrated that these genes were primarily related to the calcium signaling pathway, protein digestion and absorption, cGMP-PKG signaling pathway. In our study, we have further confirmed that the enriched calcium signaling pathway and cGMP-PKG signaling pathway were both activated during ALI (Fig. 5F). Calcium overload induces ROS accumulation, which can result in cell death (Zorov et al. 2014). Accumulating evidence suggested that disturbed calcium signals lead to increased endothelial cell permeability and inflammatory responses, resulting in endothelial dysfunction in ALI (Hao et al. 2022). Similarly, disturbed cellular calcium homeostasis is related to NLRP3 inflammasome activation in alveolar epithelial cells during LPS-induced ALI via targeting Ca^2+^/calmodulin-dependent protein kinase IV (CaMK4)(Zhang et al.2022b). In addition, our recent study revealed that activation of CaMKKII (a key factor in intracellular calcium signaling) could prevent ALI in mice by enhancing mitochondrial dynamics and mitophagy (Li et al. 2023a). cGMP-PKG signaling pathway has been reported to restrict the release of pro-inflammatory cytokines in macrophages and to diminish microvascular endothelial inflammation, both of which might participate in the development of ALI (Huang et al. 2021b; Mei et al. 2021). Reviewing the results of MeRIP-Seq and RNA-Seq conjoint analysis, we discovered that Phosphodiesterase 5 A (PDE5A) was hypomethylated but highly expressed following ALI. PDE5A is a cGMP-specific phosphodiesterase that catalyzes cGMP hydrolysis and thereby regulates the cGMP-PKG signaling pathway (Wen et al. 2020). We reasonably speculate that the cGMP-PKG signaling pathway is protectively increased during ALI, accompanied by increased PDE5A expression via m6A modification, and blocking PDE5A activity may further increase the cGMP-PKG signaling pathway to alleviate lung injury.

We have realized that there are still some unavoidable limitations to this research. First, we use lung tissues as the experimental samples in this investigation. Even though the results cannot be fully applied to humans, they do provide new insights into the underlying pathogenesis of ALI. Second, although we selected representative lung cells for research, it is indisputable that there are still other cells types (e.g. neutrophils, lymphocyte T-cells, natural killer cells) existing in lung tissue that may be involved in the development of ALI. And the interactions between different cells also need to be taken into account. Besides, despite satisfying statistical criteria, the limited sample size may lead to deviations in both the quantity and magnitude of the changed m6A modification or gene expression between groups. Therefore, future study utilizing larger sample size is needed to validate the findings. At last, whether the hub genes and enriched pathways are regulated by m6A methylation and their downstream mechanisms still require to be further explored.

## Conclusion

In summary, our findings firstly mapped m6A methylation landscape of ALI mice, and provide potential clues for the functions of m6A modification underlying the pathogenesis of ALI. In addition, we also discovered m6A methylation level was increased after ALI, accompanied by differential expressions of METTL3 and FTO. And our data further expand the expression changes of these enzymes in different lung cells. These data provide a foundation for the further investigation in the prevention and treatment of ALI.

### Electronic supplementary material

Below is the link to the electronic supplementary material.

Supplementary Material 1

Supplementary Material 2

Supplementary Material 3

## Data Availability

The datasets used and/or analysed during this study were available from the corresponding authors on reasonable request.

## Abbreviations

3′UTR: 3′ untranslated region
ALI: Acute lung injury
ALKBH5: AlkB homolog 5
ARDS: Acute respiratory distress syndrome
CaMK4: Ca2+/calmodulin-dependent protein kinase IV
CaMKII: Ca2+/calmodulin-dependent protein kinase II
CDS: Coding sequence
DEG: Differentially expressed gene
EMT: Epithelial-mesenchymal transition
Flrt3: Fibronectin leucine-rich transmembrane protein 3
FTO: Fat mass and obesity-associated protein
GO: Gene Ontology
HPMEC: Human pulmonary microvascular endothelial cell
HUVEC: Human umbilical vein endothelial cell
IGF2BP: Insulin-like growth factor 2 mRNA binding proteins
KEGG: Kyoto Encyclopedia of Genes and Genomes
LPS: Lipopolysaccharide
m6A: N6-Methyladenosine
METTL3: Methyltransferase-like 3
Pcdh17: Protocadherin 17
PDE5A: Phosphodiesterase 5 A
PPI: Protein-Protein Interaction
TLR5: Toll-like receptor 5
WTAP: Wilms tumor 1-associating protein
YTHDC1/2: YTH domain containing proteins 1/2
YTHDF: YTH-family proteins
